# Are ‘evidence-based’ cooling strategies fit-for-purpose?—Investigating the gap between guidance and behaviour

**DOI:** 10.1186/s13104-026-07820-8

**Published:** 2026-04-16

**Authors:** Mehak Oberai, Shannon Rutherford, Karlien H.W. Paas, Aaron Bach, Connor Forbes, Ella Jackman, Steven Baker, Sebastian Binnewies, Fergus K. O’Connor

**Affiliations:** 1 School of Medicine and Dentistry, Griffith University, Gold Coast, QLD Australia; 2 Griffith Institute for Human and Environmental Resilience, Griffith University, Gold Coast, QLD Australia; 3 Climate Action Beacon, Griffith University, Gold Coast, Australia; 4 School of Allied Health, Sport, and Social Work, Griffith University, Gold Coast, Australia; 5 Australian Centre for Precision Health and Technology, Griffith University, Gold Coast, QLD Australia; 6 Institute for Evidence Based Healthcare (IEBH), Bond University, Gold Coast, Australia; 7 Melbourne School of Health Sciences, University of Melbourne, Melbourne, VIC Australia; 8 School of Information and Communication Technology, Griffith University, Gold Coast, QLD Australia

**Keywords:** Adaptation, Older adults, Acceptability, Queensland, Australia

## Abstract

**Objective:**

Across two summers self-report surveys were completed by a total of 130 older adults who were trialling a digital heat early warning system in Southeast Queensland. The purpose of the project was to assess participants’ access to (accessibility), and willingness to use (acceptability) commonly promoted cooling strategies, and to further explore the reasons for reluctance to use.

**Results:**

Most participants reported access to all strategies (93–100%). Acceptability (mean response [95%CI]) was high for opening/closing windows or blinds (97 [94–100]%), fans, drinking cool water, sitting quietly, removing excess clothing (all 96 [93–99]%), cold showers (92 [87–96]%), and air-conditioning (90 [84–95] %). Conversely, hand/forearm baths (76 [69–84]%), icepacks (68 [60–76]%), dampened clothing (64 [56–72]%), foot baths (63 [54–71]%), and cold baths (49 [39–60]%) were less acceptable. Key reported barriers included messiness (*n* = 48), physical discomfort (*n* = 47), and preference for other strategies (*n* = 47). Among this sample of older adults, several widely promoted water-based cooling strategies were reported as unacceptable despite high accessibility. These findings highlight an implementation gap between public health recommendations and self-reported willingness to use such strategies, underscoring the need for co-designed heat-health interventions.

**Supplementary Information:**

The online version contains supplementary material available at 10.1186/s13104-026-07820-8.

## Introduction

Climate change, coupled with an aging global population, has led to a growing number of older adults (aged 65 or above) being more frequently exposed to extreme heat [1, 2]. Compounding this, individuals in this age group are significantly more likely to be from lower socioeconomic backgrounds [3], limiting their access to effective cooling interventions such as air-conditioning. In response, public health guidance commonly recommends low-resource personal strategies, such as electric fans (including misting fans), self-dousing, limb immersion, wetting clothing, using iced-towels and drinking cold water as effective, accessible, climate-conscious alternatives to air-conditioning [4–8]. For example, amongst other measures, the WHO recommend for individuals to “wet your skin using a damp cloth, a spray bottle, or wet clothing” to keep cool during a heatwave [6]. While laboratory and biophysical modelling investigations have identified the magnitude of physiological benefits (though in many cases, the absence of meaningful effects) for at-risk populations elicited by such interventions [9–14], emerging evidence suggests that a number of these strategies lack real-world appeal among the very populations they are intended to protect [15].

In a recent laboratory-based heat simulation study (34 °C, 77% RH) [15], older adults were asked about their willingness to use commonly promoted cooling strategies [16] when exposed to similar conditions at home without air-conditioning. Aside from utilising fans and drinking water, motivation to adopt other public-health measures including a hand and forearm bath, self-dousing or leaving home to go to an air-conditioned place, was low, where mean responses, on a scale ranging from 0 (not at all) to 10 (more than ever), were ≤ 5 [15]. However, the study did not explore why these interventions were rejected by this population. Within our individualised heat early warning system project [17] we surveyed 130 older adults, to examine self-reported accessibility and willingness to adopt (acceptability) commonly promoted cooling interventions and to explore reasons for non-use. Importantly, this study does not evaluate the physiological effectiveness of these strategies; rather, it seeks to address a critical behavioural and implementation gap between public health guidance and real-world uptake. By characterising older adults’ preferences, perceived barriers, and contextual constraints, this work aims to inform the design of heat-health messaging and interventions that are not only evidence-based, but practically acceptable and implementable within this population. It was hypothesised that older adults would have a reluctance to use water-based cooling methods.

## Methods

Participants were recruited as part of a larger project trialling a digital heat early warning system with older adults in Southeast Queensland during the 2023–2024 (*n* = 78) and 2024–2025 (*n* = 52) summers [17]. For detailed inclusion/exclusion criteria for recruiting the participants, refer to Oberai et al. [17]. Briefly however, individuals were eligible for recruitment if they (i) were aged 65 or above, (ii) were living in Southeast Queensland, (iii) were not living in a residential aged care facility, (iv) were not suffering from psychological disorders, and (v) would be home for at least 70% of the study period. During the in-home installation of the hardware components of the digital early warning system (at the commencement of each summer period) [17], participants were presented with a list of 12 different cooling strategies (Table 1) recommended in literature and public health guidance to reduce physiological heat strain [16] and were asked (i) if they had access to this strategy (yes/no); (ii) whether they would be willing to use the strategy during heat exposure (yes/no); and (iii) to explain the reason why they would not use a given strategy (For further details refer to supplementary file.docx). The survey was administered digitally via the Ethos prototype system during the installation process to the individual within the household who was enrolled in the data collection period. Participants were provided with a brief explanation of the rationale for each of the public health promoted cooling interventions prior to completing the survey. The final sample consisted of 130 participants (77 females), with a cohort median [Q1-Q3] age of 73 [69–78], and a median income range of AUD$500–649/week) (For further details refer to Table A in additional file.docx).

**Table 1:** A snapshot of accessibility and acceptability (% and 95% CI) of 12 cooling strategies with a list of reasons to not use a strategy when accessible

	Total number of times reason cited	Cold bath	Foot bath	Dampening clothes	Icepack application	Hand/ fore-arm bath	Air con-ditioning	Cold shower	Sit down quietly	Drink (cool) water	Remove excess clothes	Turn on fan	Open/ close windows/ blinds
Accessibility (% (95%CI))	**NA**	70% (62–78%)	93% (89–97%)	96% (93–99%)	94% (90–98%)	97% (94–100%)	91% (86–96%)	100% (100–100%)	100% (100–100%)	100% (100–100%)	99% (98–100%)	99% (98–100%)	100% (100–100%)
Acceptable when accessible (%(95%CI))	**NA**	49% (39–60%)	63% (54–71%)	64% (56–72%)	68% (60–76%)	76% (69–84%)	90% (84–95%)	92% (87–96%)	96% (93–99%)	96% (93–99%)	96% (93–99%)	96% (93–99%)	97% (94–100%)
Reasons for lack of willingness to implement a given intervention (n)													
Messy to perform (water dripping, risk of stains, etc.)	**43**	4	13	13	4	7	0	1	0	0	1	0	0
Physically uncomfortable to perform (too cold, too wet, etc.)	**47**	9	3	16	11	0	1	3	0	0	1	1	2
Prefer another cooling strategy	**42**	13	12	4	4	6	1	0	0	2	0	0	0
Too time consuming	**33**	11	8	2	1	3	0	4	2	0	2	0	0
Never thought of it / never tried	**30**	1	4	7	11	6	0	1	0	0	0	0	0
Unsafe (risks of slips, falls, etc.)	**16**	11	4	1	0	0	0	0	0	0	0	0	0
Too much movement required (dexterity, physical exertion, etc.)	**12**	7	2	0	1	1	0	1	0	0	0	0	0
Inconvenient	**11**	1	4	1	2	1	1	1	0	0	0	0	0
Unnecessary	**11**	0	0	5	2	4	0	0	0	0	0	0	0
Not effective /lack of benefit	**8**	0	0	4	0	4	0	0	0	0	0	0	0
Cost too much (electricity, water, maintenance, etc.)	**8**	7	0	0	0	0	1	0	0	0	0	0	0
*Other (e.g.*,* too much equipment needed*,* too noisy*,* medical reasons)*	***17***	*1*	*1*	*0*	*3*	*1*	*4*	*0*	*1*	*2*	*2*	*1*	*1*

(For details around accessibility and accesptability refer to table B in the additional file.docx)

### Statistical analyses

Descriptive analyses were conducted to explore the accessibility (number of people that had access to each strategy (x)) and acceptability (willingness to use when accessible (y)) of various cooling interventions among participants, as well as the reasons for not adopting these behaviours. Acceptability (y/x) was calculated as percentage of the trial participants who indicated willingness to use the strategy, calculated among those who reported having access to it.

Secondary correlation analysis was conducted to determine if the accessibility and acceptability of the strategies were associated with demographic variables of age (Point–biserial correlation, *r_pb*), gender (bivariate *r*), education and income (Spearman’s rho, ρ). A two-sided alpha of 0.05 was used to define statistical significance. All analyses were undertaken in SPSS statistical computing (IBM SPSS Statistics Software version: 31.0.0).

## Results

All participants completed the survey in full, providing responses to every question, resulting in a dataset with no missing values. While a majority of participants had access to the 12 cooling strategies presented (70–100%), not all interventions were deemed acceptable. Strategies that were accepted by the most respondents included: opening/closing windows/blinds (*n* = 126/130 [97%]; 95% CI 94–100%), turning on a fan (*n* = 124/129 [96%]; 95% CI 93–99%), drinking cool water (*n* = 125/130 [96%]; 95% CI 93–99%), sitting down quietly (*n* = 125/130 [96%]; 95% CI 93–99%), removing excess clothing (*n* = 124/129 [96%]; 95% CI 93–99%), taking cold shower (*n* = 119/130 [92%]; 95% CI 87–96%), and air conditioning (*n* = 106/118 [90%]; 95% CI 84–95%). In contrast, strategies such as using a hand or forearm bath (*n* = 96/126 [76%]; 95% CI 69–84%), icepack application (*n* = 83/122 [68%];95% CI 60–76%), dampening clothes (*n* = 80/125 [64%]; 95% CI 56–72%), using a foot bath (*n* = 76/121 [63%]; 95% CI 54–71%), or taking a cold bath (*n* = 45/91 [49%]; 95% CI 39–60%) were less widely accepted (For further details refer to Table B in additional file.docx).

Participants cited several barriers to use, including concerns about ‘messiness’ (n = 43), physical discomfort (n = 47), preference for alternative cooling strategies (n = 42), and the perception that the intervention was too time-consuming (n = 33). Other reasons provided included never thought of it / never tried (lack of awareness of the strategy), unsafe (risks of slips, falls, etc), too much movement required (dexterity, physical exertion, etc), inconvenient, unnecessary, not effective /lack of benefit, cost too much (electricity, water, maintenance, etc) (Table 1).

Secondary analyses revealed that gender, age, education, and income were not associated with the accessibility or acceptability of most of the cooling strategies (*p* ≥ 0.05; Table C in Additional File). However, it did reveal four statistically significant associations (see Table C, Additional File) as follows. Firstly, younger old adults were more likely to have a bath in their residence, however age did not influence whether individuals were likely to use it, or not. Secondly, individuals who had completed a lower level of education were more likely to have access to ice packs, however, this did not influence individuals’ willingness to use them. Thirdly, younger old adults were more willing to take cold showers during periods of hot weather. And lastly, older old individuals were more likely to use ice packs during periods of hot weather compared with younger older adults.

## Discussion

Our findings draw attention to the reluctance of heat at-risk older adults to utilise water-based cooling interventions commonly recommended in public health guidance [3–7]. While most respondents reported access to the cooling strategies presented, acceptability varied considerably. Simple, easy to use interventions such as adjusting windows/blinds, using fans, drinking cool water, sitting quietly, and removing excess clothing were highly accepted (> 90%). In contrast, strategies involving water immersion, additional infrastructure, or processes demanding follow-up cleaning and safety measures—such as hand/foot baths, dampening clothes, or cold baths—were less favoured (< 76%). Reported barriers included perceptions of messiness, physical discomfort, time demands, and preference for alternative methods, highlighting practical considerations that influence uptake. These findings emphasise the need for tailored heat-health communication, particularly for the older adult cohort [18].

Although many of the cooling options assessed are widely promoted in public health guidance [4–8], our findings highlight a crucial distinction between strategies recommended on the basis of tightly controlled laboratory studies [12], biophysical modelling [19] or theorised benefit for heat-at-risk populations [20], and those that our participant sample (older adults) were willing to implement during at-home heat exposure. For example, water-based methods such as hand/forearm baths, foot baths, cold baths, cold showers, and dampening clothing were not deemed readily acceptable in our study population (Table 1). Together with this, findings from the correlation analysis further suggest that with increasing age, acceptability of passive cooling strategies (e.g., ice‑pack application) tends to increase, whereas acceptability of more active strategies (such as taking a cold shower) decreases. This pattern may reflect age‑related concerns about safety, especially the heightened risk of slips and falls associated with water-based methods such as showering. Given recent Australian statistics [21] regarding injury from falls and the widescale promotion of falls risk and prevention measures, it is perhaps not surprising that participants from this age group expressed caution around use of such water-based measures.

These data highlight the need for involving at-risk populations in the development of targeted cooling interventions, ensuring that proposed strategies are feasible, culturally appropriate, and aligned with the lived realities of those most at-risk to heat [22]. Meaningful engagement with these groups can reveal barriers that are often overlooked in traditional top-down approaches and can guide the refinement of interventions to maximise both uptake and sustained use. For example, while laboratory-based investigations have identified the efficacy of the cooling interventions (dependent on environmental conditions) assessed in the current study, the effectiveness of these interventions to reduce physiological strain in community-dwelling older adults remains largely unknown. This may be best highlighted in the case of skin wetting/dampening clothes in conjunction with electric fan use. For example, while this method has been shown to provide efficacious benefit for reducing rate-pressure product (heart rate multiplied by systolic blood pressure) during short-duration heat exposures under tightly controlled laboratory conditions [12], this experimental protocol ensured that (i) participants remained seated in front of a fan for the entirety of the heat exposure, and (ii) participants skin was wetted every 5 min. Given our data indicate that participants did not deem water-based methods as acceptable cooling interventions (primarily owing to perceived messiness and water dripping, the intervention being physically uncomfortable, preference for other cooling interventions and the intervention being too time consuming), although untested, it appears unlikely that they would be willing to adhere to such a stringent protocol even if they were willing to use the intervention itself. As a result, co-developing solutions with end-users not only enhances acceptability but also likely strengthens the possibility that implemented measures will translate into measurable reductions in heat-related morbidity and mortality [23]. Testing the effectiveness of co-designed cooling interventions (inclusive of cooling protocols) presents as an important area for future research.

## Limitations

This study has several limitations. First, the sample comprised a relatively small, homogenous group of predominantly healthy older adults residing in a single subtropical region of Southeast Queensland. As such, the findings may not be generalisable to more diverse populations, including those with greater socioeconomic disadvantage, chronic disease burden, or differing cultural norms around cooling practices. Second, participation was limited to individuals enrolled in a digital heat early warning system trial, who may already be more heat-aware and health-motivated than the broader older adult population, potentially inflating reported accessibility or willingness to engage with certain strategies. Third, we did not independently verify whether participants stated willingness to use a given intervention (or not) translated into real-world adoption during heat events. Fourth, while mobility limitations were not cited as a barrier to implementation this may be resultant from our participant cohort comprising of predominantly healthy older adults of above average socioeconomic status (as measured against median population-level income) for this age group [24], whom are less likely to have mobility limitations. Further analysis should explore potential confounding effects such as physical restriction in the implementation of cooling interventions. Fifth, only one person per household was enrolled in the data collection period and therefore completed the administered survey on cooling preferences. It remains unclear whether their responses could have been influenced by other individuals living within the household (e.g., spouses). Sixth, we did not stratify responses to corresponding levels of heat exposure, and it is possible that responses may have varied in response to periods of extreme heat. Future studies should look to address these knowledge gaps. Finally, the information presented was recorded during system installation, and thus prior to any awareness of the indoor environmental characteristics of the individuals’ homes, which may influence the practicality or perceived appropriateness of different cooling behaviours. These constraints should be considered when interpreting the findings and underscore the need for future research across broader demographic, climatic, and socioeconomic settings, incorporating behavioural monitoring to assess actual uptake and effectiveness of recommended strategies.

## Conclusion

Together, our data emphasises the importance of developing heat-health guidance in partnership with the communities it is intended to serve. Evidence produced in isolation from the target population risks overlooking cultural, social, and contextual factors that shape behaviour. This emphasises the need for co-designed climate adaptation research and policy, including testing of the application of findings from laboratory studies into real life conditions, to move beyond identifying interventions that elicit physiological benefit, to understanding what works, for whom, and under what conditions. While this study examined self-reported accessibility and acceptability of commonly promoted cooling strategies, rather than physiological effectiveness or observed behavioural uptake during heat events, aside from readily implementable interventions such as opening/closing windows/blinds, using fans, drinking water, and removing excess clothing, our findings identified that most older adults did not find promoted water-based personal cooling interventions to be acceptable.

## Supplementary Information

Below is the link to the electronic supplementary material.

Supplementary Material 1.

Supplementary Material 2.

## Data Availability

The data used and/or analysed for this article are available from the corresponding author upon request. The data are not publicly available due to privacy or ethical restrictions.
