# Supplementary material for: Are ‘evidence-based’ cooling strategies fit-for-purpose?—Investigating the gap between guidance and behaviour
Source: BMC Res Notes. 2026 Apr 16;19:238. doi: 10.1186/s13104-026-07820-8 (PMC13202887; doi:10.1186/s13104-026-07820-8)
Supplement: Supplementary file 1 — Supplementary Material 1. [file 13104_2026_7820_MOESM1_ESM.docx]

# Ethos System set up (in -home installation) survey

**Field 1: User ID**- (Provided by the team)

**Field 2: Which audio alert type would you like to receive? (Choose one)**

- text-to-speech
- tone

**Field 3: What cooling strategies do you have access to?**

- Cold bath
- Hand/forearm bath
- Foot bath
- Drink (cool) water
- Cold shower
- Dampening Clothes
- Remove excess clothing
- Turn on fan
- Air conditioning
- Open/close windows/blinds
- Sit down quietly
- Ice pack application
- Other

**Field 4: What cooling strategies would you use/would you be willing to use?**

- Cold bath
- Hand/forearm bath
- Foot bath
- Drink (cool) water
- Cold shower
- Dampening Clothes
- Remove excess clothing
- Turn on fan
- Air conditioning
- Open/close windows/blinds
- Sit down quietly
- Ice pack application
- Other

**Field 5: Reason/s why you wouldn't use a cooling strategy?**

- Time consuming
- Too much equipment needed
- Too much movement required (dexterity, physical exertion, etc)
- Messy to perform (water dripping, risk of stains, etc)
- Cost too much (electricity, maintenance, etc)
- Too noisy
- Physically uncomfortable to perform (too cold, too wet, etc)
- Unsafe (risk of slips, falls, etc)
- Other

**Field 6: If other is selected, please explain**

**Field 7: Would you be interested in a follow up with a group focus discussion?**
