# Supplementary material for: Are ‘evidence-based’ cooling strategies fit-for-purpose?—Investigating the gap between guidance and behaviour
Source: BMC Res Notes. 2026 Apr 16;19:238. doi: 10.1186/s13104-026-07820-8 (PMC13202887; doi:10.1186/s13104-026-07820-8)
Supplement: Supplementary file 2 — Supplementary Material 2. [file 13104_2026_7820_MOESM2_ESM.docx]

Table A: Demographic variables of the sample

| Socio-demographic attributes |  | n (%) |
| --- | --- | --- |
| Age | 65-69 | 33 (25) |
|  | 70-75 | 36 (28) |
|  | 75-79 | 35 (27) |
|  | 80 + | 26 (20) |
| Gender | Male | 53 (41) |
|  | Female | 77 (59) |
| Education | university | 35 (27) |
|  | trade | 25 (19) |
|  | school | 66 (52) |
|  | Unclassified | 4 (3) |
| Income | Negative/Nil income | 15 (12) |
|  | $1-$149 | 2 (2) |
|  | $150-$299 | 3 (2) |
|  | $300-$399 | 2 (2) |
|  | $400-$499 | 9 (7) |
|  | $500-$649 | 24 (19) |
|  | $650-$799 | 12 (9) |
|  | $800-$999 | 19 (15) |
|  | $1,000-$1,249 | 14 (11) |
|  | $1,250-$1,499 | 6 (5) |
|  | $1,500-$1,749 | 10 (8) |
|  | $1,750-$1,999 | 4 (3) |
|  | $2,000-$2,499 | 4 (3) |
|  | $2,500-$2,999 | 4 (3) |
|  | $3,500-$3,999 | 1 (1) |
|  | $4,000 or more | 1 (1) |
| Income | Low (≤$499) | 31 (24) |
|  | Medium ($500-$799) | 36 (28) |
|  | High (≥$799) | 63 (49) |

Table B. List of 12 strategies accessible and acceptible to use by our participants.

|  | Accessibility | |  |  | Acceptable WHEN Accessible | |
| --- | --- | --- | --- | --- | --- | --- |
|  | count | % |  |  | Count | % |
| Open/close windows/blinds | 130 | 100% |  |  | 126/130 | 97% |
| Drink (cool) water | 130 | 100% |  |  | 125/130 | 96% |
| Sit down quietly | 130 | 100% |  |  | 125/130 | 96% |
| Remove excess clothing | 129 | 99% |  |  | 124/129 | 96% |
| Turn on fan | 129 | 99% |  |  | 124/129 | 96% |
| Cold shower | 130 | 100% |  |  | 119/130 | 92% |
| Air conditioning | 118 | 91% |  |  | 106/118 | 90% |
| Hand/forearm bath | 126 | 97% |  |  | 96/126 | 76% |
| Icepack application | 122 | 94% |  |  | 83/122 | 68% |
| Dampening Clothes | 125 | 96% |  |  | 80/125 | 64% |
| Foot bath | 121 | 93% |  |  | 76/121 | 63% |
| Cold bath | 91 | 70% |  |  | 45/91 | 49% |

Table C. Details of the co-relation analysis

|  |  | **Accessibility** |  |  |  |  | **Acceptability** |  |  |  |
| --- | --- | --- | --- | --- | --- | --- | --- | --- | --- | --- |
|  |  | **age (r_pb)** | **gender** | **education** | **income** |  | **age (r_pb)** | **gender** | **education** | **income** |
| **Cold bath** | **correlation** | -.204 | 0.003 | 0.012 | 0.000 |  | -0.08 | 0.103 | -0.005 | -0.042 |
|  | **p** | 0.020 | 0.969 | 0.891 | 0.996 |  | 0.45 | 0.332 | 0.964 | 0.694 |
| **Hand/fore-arm bath** | **correlation** | 0.123 | -0.057 | -0.164 | -0.002 |  | 0.050 | 0.175 | -0.085 | -0.033 |
|  | **p** | 0.162 | 0.518 | 0.066 | 0.983 |  | 0.579 | 0.050 | 0.354 | 0.711 |
| **foot bath** | **correlation** | 0.009 | 0.082 | -0.088 | 0.143 |  | 0.070 | 0.110 | -0.108 | 0.030 |
|  | **p** | 0.921 | 0.353 | 0.330 | 0.105 |  | 0.443 | 0.229 | 0.242 | 0.747 |
| **Drink (cool) water** | **correlation** | .^b^ | .^b^ | .^b^ | .^b^ |  | -0.069 | -0.085 | -0.040 | 0.113 |
|  | **p** |  |  |  |  |  | 0.438 | 0.339 | 0.659 | 0.202 |
| **cold shower** | **correlation** | .^b^ | .^b^ | .^b^ | .^b^ |  | -.256^**^ | -0.027 | -0.051 | 0.036 |
|  | **p** |  |  |  |  |  | 0.003 | 0.758 | 0.567 | 0.685 |
| **dampening clothes** | **correlation** | -0.020 | -0.085 | 0.001 | 0.017 |  | -0.017 | 0.077 | -0.075 | 0.026 |
|  | **p** | 0.822 | 0.339 | 0.989 | 0.850 |  | 0.854 | 0.393 | 0.416 | 0.773 |
| **Remove excess clothes** | **correlation** | -0.153 | -0.073 | -0.081 | -0.085 |  | 0.082 | 0.077 | 0.000 | 0.093 |
|  | **p** | 0.081 | 0.409 | 0.367 | 0.335 |  | 0.357 | 0.385 | 1.000 | 0.292 |
| **turn on fan** | **correlation** | 0.007 | -0.073 | 0.042 | 0.041 |  | 0.023 | -0.086 | 0.170 | 0.075 |
|  | **p** | 0.934 | 0.409 | 0.641 | 0.646 |  | 0.797 | 0.332 | 0.058 | 0.399 |
| **air conditioning** | **correlation** | -0.026 | -0.102 | -0.096 | 0.047 |  | 0.034 | -0.062 | 0.020 | 0.147 |
|  | **p** | 0.768 | 0.247 | 0.283 | 0.597 |  | 0.715 | 0.508 | 0.836 | 0.111 |
| **Open/close windows/blinds** | **correlation** | .^b^ | .^b^ | .^b^ | .^b^ |  | 0.055 | -0.148 | 0.002 | 0.062 |
|  | **p** |  |  |  |  |  | 0.531 | 0.093 | 0.986 | 0.485 |
| **sit down quietly** | **correlation** | .^b^ | .^b^ | .^b^ | .^b^ |  | -0.087 | -0.085 | 0.113 | 0.113 |
|  | **p** |  |  |  |  |  | 0.326 | 0.339 | 0.207 | 0.202 |
| **ice pack application** | **correlation** | -0.032 | -0.017 | -.191 | 0.074 |  | .199 | -0.035 | 0.180 | 0.116 |
|  | **p** | 0.715 | 0.847 | 0.032 | 0.403 |  | 0.028 | 0.701 | 0.051 | 0.203 |

Note: correlations with age are point-biserial correlations (r_pb), correlations with gender are bivariate correlations, and those with education and income are Spearman’s rho.

.^b^ correlations could not be computed as at least one of the variables was constant (ie., all participants had access to this strategy)
